# Gender role attitudes, awareness and experiences of non-consensual sex among university students in Shanghai, China

**DOI:** 10.1186/s12978-018-0491-x

**Published:** 2018-03-15

**Authors:** Xiayun Zuo, Chaohua Lou, Ersheng Gao, Qiguo Lian, Iqbal H. Shah

**Affiliations:** 1 Key Laboratory of Reproduction Regulation of NPFPC, SIPPR, IRD, Fudan University, 779 Laohumin Road, Shanghai, 200237 China; 2 Department of Reproductive Health and Research, World Health Organization, Geneva, Switzerland

**Keywords:** Perception, Non-consensual sex, Victimization, Gender roles, University students, China

## Abstract

**Background:**

Non-consensual sex (NCS) among young people, an important subject with public health and human rights implications, was less studied in China. This study is to investigate the NCS awareness and victimization of university students in Shanghai, China and whether they were associated with adolescent gender-role attitudes.

**Methods:**

Gender-role attitudes, awareness and victimization of different forms of NCS were examined among 1099 undergraduates (430 males and 669 females) in four universities in Shanghai using computer-assisted self-interview approach.

**Results:**

University students held relatively egalitarian attitude to gender roles. Gender difference existed that girls desired to be more equal in social status and resource sharing while more endorsed the submissiveness for women in sexual interaction than boys. They held low vigilance on the risk of various forms of NCS, with the mean score on perception of NCS among boys (5.67) lower than that among girls (6.37). Boys who adhered to traditional gender norms were less likely to aware the nature of NCS (β = − 0.6107, *p* = 0.0389). Compared with boys, higher proportion of girls had been the victims of verbal harassment, unwanted touch, fondling, and penetrative sexual intercourse. Multivariable analysis revealed that girls who held more traditional gender-role attitudes were more vulnerable to physical NCS (OR = 1.41, *p* = 0.0558).

**Conclusions:**

The weakening but still existing traditional gender norms had contributions in explaining the gender difference on the low vigilance of NCS and higher prevalence of victimization among university students in Shanghai, China. Interventions should be taken to challenge the traditional gender norms in individual and structural level, and promote the society to understand the nature of NCS better as well as enhance negotiation skills of adolescents and young people that prevent them from potentially risky situations or relationships.

## Plain English summary

With the aim to understand the gender-role attitudes, the awareness and victimization of non-consensual sex (NCS) among Chinese university students and the relationship between gender-role attitudes and NCS awareness and victimization, this study conducted quantitative analyses using data from a computer-assisted self-interview survey among university students. A total number of 1099 undergraduates aged between 18 and 24 from four universities in Shanghai were included in the analyses. Results showed that undergraduates held egalitarian attitudes to gender roles, and girls desired to be more equal in societal status and resource sharing whole more endorsed the submissiveness of women in sexual interaction than boys. They held low awareness on the risk of NCS and such awareness among boys was even lower. Compared with boys, higher proportion of girls had been the victims of verbal harassment, unwanted touch, fondling, and penetrative sexual intercourse. The relationship between gender-role attitudes and NCS awareness and victimization varied between gender: boys who adhered to traditional gender norms were less likely to aware the nature of NCS, while girls who held more traditional gender-role attitudes were more vulnerable to physical NCS. Such findings suggested gender-sensitive interventions should be developed to question the traditional gender norms in the individual and structural level, and promote adolescents to understand the nature of NCS better as well as keep away from potentially risky situations or relationships.

## Background

Non-consensual sex (NCS), interchangeably used as sexual abuse, sexual violence and sexual coercion, in general was operationalized to encompass a range of behaviors including unwanted penetrative sex, attempted rape, unwanted touch, as well as non-contact forms of abuse such as, verbal harassment or forced viewing of pornography. These acts may include any coercive situations that the victims lack of realistic choices available to prevent or redress the situation, for example physical violence, threats, intimidation, emotional manipulation and deception [1]. The issue of NCS among adolescent and young people is an important subject with not only public health implications, but also a violation of human rights with legal concerns as well. Significant numbers of young people, particularly girls and young women but also boys and young men, are exposed to NCS around the world places [2, 3]. Meanwhile, young people may be less equipped than adults to avoid incidents of NCS and may have fewer choices available to them to cope with such incidents. A growing number of studies show that experience of NCS during childhood and adolescence often adversely affects their subsequent psychological, behavioral, and reproductive outcomes [4–8].

A large body of research on adolescent NCS, with unspecified definitions and varying in research population and research methods have produced varied findings. For example, a wide range of prevalence from less than five to over 50 % is noted among adolescents from multiple countries [1, 9, 10]. Most surveys that report on this subject ask a fairly general question, usually on the line of “Have you even been forced to engage in sex?”. Diversity of evidence obtained from such measurement might be partially attributed to the dynamic nature of young people’s interpretations of NCS. For instance, young people who submit to the pressure of a partner’s demands for sex as an expression of commitment may not respond affirmatively to a general question on “forced” sex. Marston found that young unmarried girls in Mexico identified sexual experience as non-consensual only if the perpetrator was someone with whom they didn’t have a romantic relationship [11]. Young people’s interpretation of NCS could be influenced by the sociocultural context and legal framework that young people live in. In some settings, early marriage is encouraged and young women are socialized to believe it is their duty to accept the sex from their husband even if they are not willing to do so [12]. It is, therefore, quite important to provide precise and detailed interpretation of NCS and obtain youth’s interpretation on them when conducting research on NCS among young people in particular sociocultural context.

NCS is influenced by multiple risk factors existing at different levels, from the individual to the community and societal level. Scholarly efforts have highlighted the role of gender norms. Gender norms, as defined by The World Health Organization, refer to beliefs about women and men, boys and girls that are passed from generation to generation through the process of socialization. They change over time and differ in different cultures and populations. Gender norms lead to inequality if they reinforce mistreatment of one group or sex over the other, or differences in power and opportunities [13]. UNAIDS reported at the macro level that sexual violence against women appears to be more common in settings where gender roles are rigidly enforced and where masculinity is associated with toughness and dominance while femininity with submissiveness [14]. Some empirical research in developed countries suggested the effects of individual attitudes to gender norms: young women who explicitly endorsed traditional gender beliefs were particularly disempowered during sexual interactions and at higher risk for NCS [15, 16]; male adolescents who supported traditional gender norms and beliefs in the inferiority of women and girls were more likely to report sexual violence and perpetration of intimate partner violence [17, 18]. Neverthless some studies have failed to find significant association between NCS victimization and individual gender-role attitudes [14, 19, 20]. However, to our knowledge, no such studies on gender-role attitudes and victimization or perpetration of NCS have been conducted in China with specific cultural context on gender norms. Such findings would be extremely relevant to informing future NCS research and practice efforts.

China, with a traditional Confucian culture influenced deeply for thousands of years, has initiated many adolescent sexual and reproductive health research and programs. Most of these assume that young people’s sexual experience, except rape, is voluntary. Few studies have considered the issue of NCS, however, in fairly general and ambiguous way, asking questions like “Have you ever been forced to engage in sex?” [21, 22]. There is no particular research to examine views on and prevalence of different kinds of NCS among young people, while anecdotal accounts suggest such incidents are not rare. The dearth of such evidence can probably compromise the beneficial effects of adolescent sexual and reproductive health promotion programs that remain largely uninformed of the evidence of appropriate strategies and interventions.

The traditional Confucian culture in China has strict doctrines linking unequal gender stratification and distribution of power and resources by its core of “Three obedience” (*san cong*) particularly for women, namely women subordinate to men in every stage of life: daughters to their fathers, wives to their husbands, and in widowhood, to their sons. Female were not allowed to go “out” to school but were expected to stay home learning skills of housework to raise family after getting married [23]. A woman should always be modest and submissive in manner, otherwise it could damage the reputation of her family, and the man was expected to be responsible and gentlemanlike. Chastity was particularly required of women that they should not only remain virginal until marriage but also maintain absolute fidelity toward their husbands, whether he was alive or dead. Women should be kept passive and sexually innocent in relationship with men. This was not the case for men. With the “open” policy in effect since the late 1970s, considerable changes in economy and globalization of social culture have taken place, and the traditional Confucian ideals of gender roles have been weakening gradually [24]. There is rapid rise in percentage of women getting high level of schooling and entering the labor market. More men and women are working in less traditional careers and sharing family and household responsibilities. Increasing number of adolescents and youth are now engaged in premarital sex [25]. However, the current attitudes to gender roles among adolescents were not known when they are witnessing these changes.

Thus, to better informing the programs and policies in China, the current study objectives include examining the awareness and victimization of NCS among a sample of university students, examining their attitudes to gender roles, and demonstrating evidence of associations between attitudes to gender roles and NCS awareness and victimization.

## Methods

### Sample and procedures

Four universities in Shanghai were selected purposively in 2009 to represent different academic rankings. From four types of disciplines, i.e. engineering, science, liberal arts and arts (including music, painting, movie etc.) in each selected university, one department, and then third-year students were randomly selected to achieve the sample size of male and female students. Only the third-year students were chosen due to two considerations – anticipation of more sexually active (consensual or non-consensual) adolescents among higher grade students and the fact fourth-year students are usually out of school preparing for graduation field work and, therefore, difficult to reach. The unequal sample size among male and female students was determined by the prevalence of non-physical and physical childhood sexual abuse with gender difference among adolescents suggested by a study conducted in China [26]. Totally 1099 students (430 males and 669 females) aged 18–24 years (mean age: 21.5 years) were recruited and voluntarily participated in the study with informed consent (accounting for 69.1~ 88.8% of students in each selected department). Four male and 3 female students refused to participate in the study. Nearly all of the students (99.8%) were unmarried and majority of them (88.1%) lived in the dormitory. One-third of them had lived in rural areas before entering the university. About 43% and 38.9% of respondents respectively reported their parent’s educational level as high/vocational/technical school and college or above. Most of them (88.4%) had relatively good feeling about their family life and about one in two regarded their campus life as good. About 29.5% and 16.6% of male and female subjects reported they had experienced sexual intercourse, including consensual and non-consensual.

After obtaining their informed consent, university students were organized to fill the questionnaire in computer classrooms during the lunch break. With the computer-assisted self-completion interview (CASI), each participant read and answered questions on a computer without interposition of interviewers, who were trained to assist respondents, when necessary, in understanding questions and in handling any emergency during the survey. Information about respondents’ views on and experience of NCS, relevant feelings, reactions and consequences, their attitudes to gender roles, among other topics was collected anonymously. In addition to providing anonymity, CASI allows for programmed consistency checks and skip patterns that reduce errors, and it eliminates the need for an additional data entry step after the assessment is completed. All study procedures were approved by the institutional review board of Shanghai Institute of Planned Parenthood Research (SIPPR) and WHO Research Ethics Review Committee.

### Measures

#### Attitudes to gender roles

Six typical questions were asked to ascertain respondents’ views about the gender division of roles in family, in social resource allocations and in sexual relations: (1) “household should be led by men”, (2) “in general, boys should get more schooling than girls”, (3) “when jobs are scarce, men should have more right to job than women”, (4) “women should have same opportunities as men in leadership”, (5) “it is acceptable for a husband to beat his wife in some situations”, (6) “a woman should not be the first to show a man that she likes him”. For each question, the response was measured on 3-point ordinal scale from disagreed (0), uncertain (1), and agreed (2). We reversed the fourth question, and then summed students’ responses to get an index with the maximum of 12. The higher the score, the traditional the students’ values and the less egalitarian gender-roles they held. The median of the composite index was 2.

#### Awareness on NCS

We adopted the definition of NCS provided by Jejeebhoy, et al. [1]. Accordingly, nine episodes on different kinds of NCS were listed, containing verbal harassment via communication technologies (short message, telephone and on the Internet, etc.), forced viewing of pornographic material or video, forced exposure to exhibitionism, watched secretly while changing clothes or in similar situations, secret pictures or flashing disseminated on the Internet without permission, sexually suggestive talking, unwanted touch, foundling, as well as forced penetrative sex through threats, intimidation, emotional manipulation, deception, material and non-material incentives. Respondents were asked whether each mentioned episode was NCS and responses were scored “1” if they answered in affirmative and “0” if they answered negatively or “didn’t know”. These scores were summed up for the composite index (maximum score was 9) to reflect the respondent’s overall perception of NCS.

#### Experience of NCS

Likewise, to avoid ambiguity, different forms of NCS aforementioned were separately listed to measure whether respondents had experienced such incidents. A typical question on one form of NCS was “have you ever encountered someone touching the private parts of your body or someone touching you with his/her private parts of body that made you feel uncomfortable or embarrassed?”. We tried to get the prevalence of each form of NCS to make our results comparable to those from other studies.

### Control variables

The demographic variables – gender, age, discipline, residence, feeling about family atmosphere and current campus life, and parent’s educational level were included in the multiple regression analysis as control variables. All these variables, including parent’s educational level were self-reported by university students. Parent’s educational level was determined by the higher level of his/her father and mother to partially reflect the socioeconomic status of respondent’s family.

### Analysis

The original data collected via the electronic questionnaire were managed by Microsoft Access, which can be transferred into SAS dataset. The transformed data then were checked and analyzed using SAS statistical package 9. As gender was an important factor in understanding sexual issues, all analyses were stratified by gender (male, female). Preliminary analysis used chi-square, t-test and ANOVA to examine the gender difference of attitudes to gender roles, and perception and experience of NCS. Then, multiple-regression (general linear model) and logistic regression were used to determine the influence of gender-role attitudes on experience of NCS among university students, adjusting for the effects of demographic covariates.

## Results

### Attitudes to gender roles

The responses supporting traditional gender roles ranged from 1% to 47% (Table 1). More than 20% of students endorsed the unequal position in family and social resource allocation between males and females. Only 2.9% of students accepted the physical violence against woman by her husband. Compared with girls, higher percentage of boys favored the resource sharing and power dominance of males. However, in sexual relations, higher proportion of girls accepted the traditional dogma of submission for females than did boys. The mean score of gender-role attitudes was higher for boys than for girls (3.59 vs 2.10), which meant boys held more gender-unequal attitudes. Even within groups of similar characteristics, such gender difference was found consistently.

**Table 1:** Distribution of respondents’ attitudes to gender roles, by sex of the respondent (%)

Items of traditional gender roles	Total (*n* = 1099)	Male (*n* = 430)	Female (*n* = 669)
Household should be led by men.			
Disagree	46.95	26.98	59.79
Uncertain	21.93	25.81	19.43
Agree	31.12	47.21	20.78
In general, boys should get more schooling than girls.			
Disagree	62.24	55.81	66.37
Uncertain	16.56	20.93	13.75
Agree	21.20	23.26	19.88
When jobs are scarce, men should have more right to a job than women.			
Disagree	64.15	43.95	77.13
Uncertain	15.20	24.19	9.42
Agree	20.66	31.86	13.45
Women should have same opportunities as men to leadership positions.			
Disagree	6.01	10.00	3.44
Uncertain	9.83	18.60	4.19
Agree	84.17	71.40	92.38
It is acceptable for a husband to beat his wife in some situations.			
Disagree	91.08	81.86	97.01
Uncertain	6.01	12.79	1.64
Agree	2.91	5.35	1.35
A woman should not be the first to show a man she likes him.			
Disagree	71.43	81.16	65.17
Uncertain	22.11	16.05	26.01
Agree	6.46	2.79	8.82

All were significant at *p* < 0.001compared between male and female respondents

### Perception of NCS

For ease of description, the median splits were performed on the score of gender-role attitudes to categorize respondents as relatively traditional (score > 2) or egalitarian (score < =2) in their gender ideology. The analysis revealed students’ low awareness of non-consensual sex in terms of the general perception score, with 5.67 as the mean score of recognition among boys, a little lower than among girls (6.37). Males who held more egalitarian attitudes had higher composite score of the recognition than those who held more unequal attitudes (6.02 vs. 5.45, *p* = 0.048). While among girls, the score of general recognition were of no significant difference between those favoring egalitarian or unequal attitudes (6.44 vs. 6.24, *p* = 0.378).

The proportion of male and female students who were aware of coercion on typical scenarios varied between 48.1~ 93.4%, with higher recognition of the kinds of physical sexual coercion including unwanted touch, fondling and penetrative sex (Table 2). Compared with girls, lower proportion of boys viewed events like forced watching of pornographic materials, disseminating private pictures without permission, unwanted fondling, and forced penetrative sex as NCS. Among boys, higher proportion of those who held egalitarian gender-role attitudes viewed unwanted touch, fondling and penetrative sex as non-consensual than those holding unequal attitudes, while the two groups of boys held no different views on the non-physical coercion. Among girls, however, no associations between gender-role attitudes and views on all forms of NCS were found.

**Table 2:** Proportion of students who perceived the risk of NCS, grouped by sex of the respondent and gender-role attitudes (%)

Items on NCS	Male			Female		
	Total	Gender-role attitudes Score		Total	Gender-role attitudes Score	
		<=2 (*n* = 164)	> 2 (*n* = 266)		<=2 (*n* = 438)	> 2 (*n* = 231)
Score (mean)	5.67	6.02	5.45*	6.37^#^	6.44	6.24
Verbal harassment via communication technology	48.14	50.61	46.62	51.87	52.28	51.08
Forced watching porn	57.44	62.20	54.51	65.32^#^	66.44	63.20
Forced exposure to exhibitionism	60.70	64.63	58.27	76.38^#^	78.08	73.16
Watched secretly while changing clothes or in similar situations	54.42	56.10	53.38	60.09	60.50	59.31
Disseminating secret pictures/flash without permission	53.26	53.05	53.38	61.73^#^	62.10	61.04
Sexually suggestive comments	48.37	52.44	45.86	53.96	54.11	53.68
Unwanted touch/frottage	75.81	82.32	71.80 *	84.45	85.16	83.12
Unwanted fondling	80.00	87.20	75.56 *	89.54^#^	90.41	87.88
Unwanted penetrative sex	88.60	93.29	85.71*	93.42^#^	94.52	91.34

^#^significant at *p* < 0.01compared between gender

*significant at *p* < 0.05compared between two groups holding different gender-role attitudes within boys or girls

### Experience of NCS

Most frequently mentioned experience of NCS was unwanted touch, reported by 27.2% and 63.3% respectively among boys and girls. About 50% of all boys and girls reported experiencing verbal harassment via short message, telephone and on the Internet. Also, about 20% of boys and 33.9% of girls had encountered sexually suggestive comments. Lower rate, about 7.7% of boys and 13.9% girls reported experiencing unwanted fondling, and less than 2% of boys and about 8% of girls reported attempted forced penetrative sex. About 1.9% and 5.2% of boys and girls respectively had encountered forced sexual intercourse, which accounted for 6.3% and 31.5% of sexually active boys and girls respectively (Table 3). We also found association of gender-role attitude and different forms of NCS experience. Among boys, lower percentage of those who held egalitarian attitudes to gender roles reported having experienced sexually suggestive comments and unwanted fondling. Lower percentage of girls holding egalitarian attitudes reported being victims of unwanted touch and forced penetrative sex.

**Table 3:** Percentage distribution of students experiencing different forms of NCS, grouped by sex of the respondent and gender-role attitudes (%)

Forms of NCS	Male			Female		
	Total	Gender-role attitudes Score		Total	Gender-role attitudes Score	
		<=2 (*n* = 164)	> 2 (*n* = 266)		<=2 (*n* = 438)	> 2 (*n* = 231)
Verbal harassment via communication technology	47.67	48.17	47.37	49.48	50.46	47.62
Sexually suggestive comments	20.00	15.24	22.93*	33.93^#^	32.42	36.80
Unwanted touch/frottage	27.21	26.22	27.82	63.23^#^	60.50	68.40*
Unwanted fondling (*n*)	7.67 (33)	4.27 (7)	9.77* (26)	13.90^#^ (93)	13.01 (57)	15.58 (36)
Attempted rape (*n*)	1.40 (6)	0.61 (1)	1.88 (5)	8.07^#^ (54)	6.62 (29)	10.82 (25)
Completed forced sex (*n*)	1.86 (8)	1.22 (2)	2.26 (6)	5.23^#^ (35)	3.42 (15)	8.66^*^ (20)

^#^significant at *p* < 0.01compared between gender

*significant at *p* < 0.05compared between two groups holding different gender-role attitudes within boys or girls

### Association between gender role attitudes and NCS awareness and victimization

Table 4 presents the results from General Linear Model (GLM) of students’ awareness of NCS, with the score of awareness as the dependent variable. The independent variables included gender-role attitudes, and the demographic variables as covariates. Gender-role attitudes were negatively associated with the perception of NCS among boys (β = − 0.6107, *p* = 0.0389), which meant boys who had more traditional attitudes of gender roles were less likely to recognize the coercion of specific events. However, gender-role attitudes were not significantly associated with perceptions of NCS among girls, controlling for the background characteristics included in the analysis.

**Table 4:** Results of multiple regression (generalized linear model) assessing the influencing factors of perception of NCS, by sex of the respondent

Variables	Male		Female	
	Coefficient (β)	*p-*value	Coefficient (β)	*p-*value
Gender-role attitudes	−0.6107	0.0389	−0.1269	0.5642
Discipline ^a^				
Literal arts	−0.0398	0.9312	0.7158	0.0104
Science	0.4269	0.3902	0.5256	0.1665
Engineering	0.2781	0.5099	1.0271	0.0004
Residence before entering university ^b^	−0.0851	0.8005	−0.3830	0.1131
Parent’ s highest education	0.4002	0.0652	0.1732	0.2665
Feeling about family atmosphere	0.1329	0.7185	0.3592	0.1509
Feeling about campus life	−0.0370	0.8663	− 0.1521	0.4021

^a^arts as the reference

^b^rural area as the reference

Table 5 shows results of students’ experience of physical sexual coercion using logistic regression, controlling for demographic covariates. Here, for efficiency of analysis and ease in interpretation, we merged together all those who reported having had experienced either unwanted touch, fondling, attempted or actual penetrative sex as one category of having experienced physical NCS. The positive association between traditional gender-role attitudes and experience of physical NCS was not found among boys, but marginally significant (OR = 1.41, *p* = 0.0558) among girls. It seemed that girls who held more traditional gender-role attitudes were more likely to be the victims of physical NCS. We also analyzed the association between gender-role attitudes and experience of verbal NCS (face-to-face and via technology together or respectively) and no associations were found (data not shown).

**Table 5:** Results of logistic regression assessing the influencing factors of physical NCS experience, by sex of the respondent

Variables	Male		Female	
	Coefficient (β)	*OR (95%CI)*	Coefficient (β)	*OR (95%CI)*
Gender-role attitudes	0.0870	1.09 (0.70–1.69)	0.3440	1.41 (0.99–2.01)
Discipline ^a^				
Literal arts	−0.0132	0.75 (0.38–1.47)	0.0487	0.99 (0.63–1.54)
Science	−0.4091	0.51 (0.24–1.08)	0.0701	1.01 (0.55–1.84)
Engineering	0.1505	0.88 (0.48–1.62)	−0.1790	0.79 (0.50–1.24)
Residence before entering university ^b^	−0.1619	0.85 (0.52–1.39)	0.4938	1.64 (1.13–2.37)
Parent’ s highest education	−0.0814	0.92 (0.67–1.27)	− 0.0043	0.99 (0.78–1.27)
Feeling about family atmosphere	0.3581	1.43 (0.86–2.39)	0.6343	1.89 (1.18–3.01)
Feeling about campus life	−0.0779	0.92 (0.67–1.28)	− 0.0968	0.91 (0.68–1.21)

^a^arts as the reference

^b^rural area as the reference

## Discussion

To promote the awareness of the problem of NCS, adolescent health and rights advocates are calling for more research and an increased emphasis on its prevention. It is essential to better understand adolescents’ perceptions of NCS to effectively address it, as self-disclosure is critical in identifying such events. This study, involving a range of NCS, was the first study of its kind in China that examined university students’ perception of NCS. It found most unwanted physical contacts were viewed as abusive by majority of university students, while non-physical sexual violence was viewed as such by half of them. Their perception of NCS was, therefore, affected by the intrusiveness of the sexual act, and physical contact seemed to be the recognized threshold. This finding is consistent with those from US and Korean studies [27, 28]. In China, any act of rape against women or girls has been included in the law with relatively clear definition and accountability of perpetrators. However, sending pornographic message, indecent behavior and intentionally exposing one’s body to others, and sexual harassment against women were ambiguously included in different laws and regulations since 2005 without clear penalty regulations. Such legalization status might limit the attention of the whole society to non-physical sexual violence and restrict the provision of education and further services for victims or survivors. This could partly explain the lower awareness on non-physical sexual violence among university students.

As found in other studies, females were much more likely to experience NCS than males. However, NCS rates were non-trivial rates for male university students. This pointed out the necessity that prevention programs reach out to men as potential victims. The most frequently reported form of NCS among university students was unwanted touch. However, unwanted touch is often overlooked by the public mainly owing to its seemingly less intrusiveness. The extent of negative effects in the aftermath of unwanted touch as compared to more intrusive NCS needs to be further studied. The forced penetrative sex was found to be higher (31.5%) among sexually active female students, compared to what has been reported in other studies conducted in Chinese universities [29]. The differences in the phrasing of questions and the types of NCS used in different studies may in part explain the divergence in findings. The emergence of sexual harassment through short message, telephone or the Internet relevant to today’s adolescents is serious because of the growing number of adolescents using these technologies, which was also indicated by other studies [30, 31]. The wide application of information technology and the Internet endow sexual harassment with more versatile and hidden forms, requiring an urgent attention by policy makers and sexual education or service practitioners.

The study documents the transition from traditional gender norms to support for the egalitarian gender roles among university students in Shanghai. Over half of the students supported equal gender roles, while some were undecided. Gender difference was found in the adherence of the egalitarian gender roles. Girls expressed to be more equal in social status and resource sharing, while persistently showing the traditional gender role of female submissiveness in sexual interactions. This study found female university students who held traditional gender-role attitudes were more likely to be victims of NCS. Valuing deference and subordination could affect women’s behaviors generally as well as during sexual encounters. Wigderson and Katz found college women invested in feminine deference were less assertive in refusing unwanted advances, and being less assertive in turn appeared to increase the risk of sexual assault [32]. In addition, as the still entrenching traditional gender norms expected women to be virgin and uninformed about sexual matters before marriage, young girls were less likely to have enough sexual knowledge and negotiation skills, and hence were unable to express their consent to sex or to resist sexual advances. This study also found the gender differences in the interpretation of NCS and the negative influence of traditional gender roles on perception of NCS among male university students. Men who held dominant masculine norms were more inclined to justify the sexual violence, especially when the violence toward women and girls. Extensive research has documented that men with more traditional gender role ideologies are significantly more likely to report sexual coercion and relationship violence [17, 18, 33, 34], and there has been emphasis on challenging traditional masculine ideologies in the programs involving boys and men in other countries [35–37]. This study also pointed a critical need to challenge the traditional masculine norms in China.

Gender-role attitudes had no impact on NCS experience among male students. As most of male students (50–58%) encountered male-to-male sexual coercion while majority of female students (80–98%) encountered heterosexual coercion, traditional gender norms do not adequately explain the experience of male-to-male sexual coercion. Further research should therefore explore the impact of traditional gender norms on young men’s role as perpetrators and the underlying factors influencing the homosexual sexual violence among college males. Besides, no association was found between gender-role attitudes and overall perception on NCS among female students. It is possible that gender norms have several dimensions and the scale used in this study didn’t capture meticulously the ideologies as perceived by today’s university students that affect their awareness on NCS. Therefore, further research should investigate the potential dimension of gender role perceptions among young university students in terms of how these perceptions might be related to vigilance of NCS risks.

The results of the current study point out the need to not only improve the policy and legislation on kinds of NCS, but also educate the public on the variety of situation which constitute NCS. Such actions could significantly boost the identification and support services for the victims and survivors. Specifically, relevant policies and legislation should provide explicit definition on each kind of NCS and provide operable and detailed punishment regulations. Education programs should serve to increase the adolescents’ vigilance of “danger” signs, especially when the case of sexual coercion does not fit the stereotype. Meanwhile, education programs should increase the NCS awareness of relevant professionals, emphasize that appropriate actions are taken when professionals recognize such cases, and thus increase the probability that adolescent and youth would receive potential mental, health, and legal support. The possibility that males could be potential victims of NCS should not be overlooked in the prevention and support services and programs. The findings also offer a strong justification to question the weakening but still entrenched traditional gender norms, both passive femininities and dominant masculinities, that favor coercion against women and have devastating impact on women’s ability to assert themselves in sexual encounters and relationships. Young men need to better understand women’s rights and learn skills to resolve conflicts. Young women should be empowered with negotiation and self-protection skills that would build their self-esteem and prevent them from potentially risky situations or relationships. Challenging harmful gender norms and unequal power between males and females requires going beyond individual level efforts to challenging gender inequalities at the structural level [38]. Support from the whole society, including family, school, media, government institutions and law enforcement, should be mobilized to eliminate the harmful gender norms and discriminatory practices.

Several limitations of the study are noteworthy. First, only six items were included in our exploratory study to reflect Confucian-based gender norms. Our conclusion for the effect of gender norms are considered tentative and appropriate scale to capture gender-role ideologies perceived by today’s adolescents should be developed in future research. Second, this study didn’t collect information regarding respondents as perpetrators for the reason that respondents would have feared punishment or stigma and were less likely to report it. Therefore, the effects of traditional gender-role attitudes on acts of perpetrators couldn’t be explored. Third, data were based on self-reporting. Although computer-assisted self-interviewing method has proved to reveal higher prevalence rates for sensitive and stigmatized behaviors [39], recall bias cannot be ruled out. Fourth, the participants were recruited from four universities in Shanghai. The social norms and environment in these universities may have influenced the knowledge and information available to students as well as their assessment of harassment. Therefore, the results from this study cannot be considered to be representative of Chinese youth. Finally, given that the study was cross-sectional, causal interpretation of relationship cannot be drawn. For these limitations, results from this study should be considered exploratory in nature though they provide important insights on gender-role attitudes, perceptions and experience of NCS among university students in Shanghai.

## Conclusion

This study found university students in Shanghai, China held relatively egalitarian attitude to gender roles and low awareness on NCS, but faced relatively high risk of being the victim of NCS. The weakening but still existing traditional gender norms had negative effects on the vigilance of NCS among male students and victimization among female students. The present study suggested the need of challenging the harmful gender norms both in individual and structure level while promoting policy and legislation as well as education and support programs on NCS, in order to help adolescents and young people to understand the nature of NCS better and enhance sexual self-efficacy and negotiation skills that prevent them from potentially risky situations or relationships.

## Abbreviations

95%CI: 95% confidence interval
NCS: Non-consensual sex
OR: Odds ratio
